# Diagnostic Accuracy of Corneal and Epithelial Thickness Map Parameters to Detect Keratoconus and Suspect Keratoconus

**DOI:** 10.1155/2023/6677932

**Published:** 2023-10-06

**Authors:** Abdelrahman Salman, Cosimo Mazzotta, Obeda Kailani, Marwan Ghabra, Rana Omran, Ashraf Armia Balamoun, Taym Darwish, Rafea Shaaban, Hala Alhaji

**Affiliations:** ^1^Department of Ophthalmology, Tishreen University, Latakia, Syria; ^2^Departmental Ophthalmology Unit, AUSL Toscana Sudest, Siena, Italy; ^3^Ophthalmology School, University of Siena, Siena, Italy; ^4^Siena International Crosslinking Centre^®^, Siena, Italy; ^5^Faculty of Life Sciences and Medicine, King's College London, London, UK; ^6^Whipps Cross University Hospital, Leytonstone, London, UK; ^7^Eye Surgical Hospital, Damascus, Syria; ^8^Watany Eye Hospital (WEH), Cairo, Egypt; ^9^Watany Research and Development Centre, Cario, Egypt; ^10^Ashraf Armia Eye Clinic, Giza, Egypt; ^11^Tartous University, Tartous, Syria

## Abstract

**Aim:**

To establish the diagnostic accuracy of corneal and epithelial thickness measurements obtained by spectral-domain optical coherence tomography (SD-OCT) in detecting keratoconus (KC) and suspect keratoconus (SKC).

**Methods:**

This retrospective study reviewed the data of 144 eyes separated into three groups by the Sirius automated corneal classification software: normal (N) (*n* = 65), SKC (*n* = 43), and KC (*n* = 36). Corneal thickness (CT) and epithelial thickness (ET) in the central (0–2 mm) and paracentral (2–5 mm) zones were obtained with the Cirrus high-definition OCT. Areas under the curve (AUC) of receiver operator characteristic (ROC) curves were compared across groups to estimate their discrimination capacity.

**Results:**

ROC curve analysis revealed excellent predictive ability for ET variables: minimum (Min) ET (0_2), minimum-maximum (Min-Max) ET (0_2), superonasal-inferotemporal (SN-IT) ET (2_5), Min-Max ET (2_5), and Min ET (2_5) to detect keratoconus (AUC > 0.9, all). Min-Max CT (0_2) was the only CT parameter with excellent ability to discriminate between KC and N eyes (AUC = 0.94; cutoff = ≤−32 *μ*m). However, both ET and CT variables were not strong enough (AUC < 0.8, all) to differentiate between SKC and N eyes, with the highest diagnostic power for Min-Max ET (2_5) (AUC = 0.71; cutoff = ≤−9 *μ*m) and central corneal thickness (CCT) (AUC = 0.76; cutoff = ≤533 *μ*m).

**Conclusion:**

These results demonstrate that OCT-derived CT and ET are able to differentiate between KC and N eyes, with a high level of certainty. However, Min-Max ET (2_5) was the parameter with the highest ability to detect suspect keratoconus.

## 1. Introduction

Keratoconus (KC) is a progressive corneal disease characterized by thinning of the central or paracentral portion of the cornea resulting in irregular astigmatism and subsequent visual impairment [1]. Several studies from the Middle East have demonstrated a high prevalence rate of KC [2–4]. KC is typically characterized by thinning of the stroma with the largest thickness changes in the 5 mm zone [5].

Currently, multiple diagnostic modalities are available to measure total corneal thickness and corneal epithelial thickness (ET), including very high-frequency (VHF) digital ultrasound, confocal microscopy, and anterior segment optical coherence tomography (AS-OCT) [6–8]. AS-OCT is a noncontact, high-resolution imaging technique that allows precise delineation of corneal surfaces and layers.

Among the various parameters proposed by Randelman et al. to predict the risk of postlaser in situ keratomileusis (LASIK) ectasia, unrecognized early-stage keratoconus is considered a vital risk factor of this disease [9]. Several corneal topographic-based parameters have demonstrated good accuracy for detecting manifest KC [10–12]. However, their accuracy in detecting suspect keratoconus (SKC) is limited [13]. In early KC, corneal epithelial remodelling can mask the stromal irregularities, resulting in false-negative topographic findings [14]. However, this compensatory epithelial thickness modulation can be an important diagnostic parameter to detect early or subclinical KC.

Our previous studies have demonstrated the diagnostic value of topographic, tomographic, and higher-order aberration parameters utilizing the Sirius (Costruzione Strumenti Oftalmici, Florence, Italy) corneal tomography in detecting SKC and KC [13, 15]. Evaluating the changes that occur at the level of corneal epithelium may provide additional information for the detection of SKC and prevent postoperative LASIK ectasia. The aim of this study was to investigate the diagnostic ability of CT and ET parameters obtained with the Zeiss Cirrus 5000 HD-OCT (Carl Zeiss Meditec, Dublin, CA, USA) in differentiating between SKC, KC, and normal (N) corneas.

## 2. Materials and Methods

### 2.1. Study Participants

This was a retrospective, case-controlled study. The study adhered to the tenets of the Declaration of Helsinki, and Institutional Review Board approval was obtained (approval number TUH-06023). All participants were at least 18 years old and provided written informed consent from the Department of Ophthalmology of Tishreen University Hospital, Latakia, Syria.

Artificial intelligence was used to classify the participants into three groups (N, SKC, and KC) utilizing the Sirius Corneal Navigator automated corneal classification software. The Sirius classification algorithm (Phoenix) had the highest accuracy in detecting KC (91.24%) and SKC (88.68%) [16].

The data from a single eye was randomly included if both eyes were positive for KC or SKC. Only data from one eye was included from N patients. Patients were recruited consecutively, and all subjects meeting the inclusion criteria were included in the study.

Patients who had undergone LASIK with 3 years of follow-up and no documented evidence of ectasia or postoperative complications were included in the N group. Eyes in the N group had a normal Sirius software classification, and baseline preoperative corneal topography data did not reveal findings suggestive of KC, such as focal or inferior steepening of the cornea or central keratometry greater than 47.0 D in either eye. All eyes in the N group had a corrected distance Snellen (feet) visual acuity of 20/20 or better.

Diagnosis of SKC and KC was confirmed by an experienced cornea specialist (AS) with more than 10 years of experience. The SKC group was characterized by a positive Sirius software indicator, absence of clinical (keratometric, retinoscopic, or biomicroscopic) signs of KC in either eye, and corrected distance visual acuity (CDVA) of 20/20 or better.

The diagnosis of KC was made if there was (a) an irregular cornea determined by distorted keratometry mires and/or distortion of the retinoscopic reflex; or one of the following slit-lamp findings: Vogt striae, 2 mm arc of Fleisher ring, apical thinning, Munson's sign, Rizutti's sign, or corneal scarring consistent with KC, in addition to (b) a positive Sirius software indicator.

Patients were excluded if they had corneal hydrops, previous corneal or ocular surgery (such as cataract, glaucoma, corneal collagen cross-linking, excimer laser surgery, intrastromal corneal rings, and phakic intraocular lens), a history of corneal oedema, any pathology of the cornea and anterior segment that may confound with corneal parameters (dry eye disease, keratitis, glaucoma, uveitis, and Fuch's dystrophy), autoimmune disease, breastfeeding, or pregnancy. Contact lens wearers in the preceding 3 months were also excluded.

Ocular assessment included auto-refracto-keratometry (SEIKO CO, GR-3500KA, Japan), uncorrected distance visual acuity (UDVA), corrected distance visual acuity (CDVA), slit-lamp biomicroscopy, Goldmann applanation tonometry, fundoscopy, and Placido/Scheimpflug-based Sirius corneal imaging.

**Figure 1:**
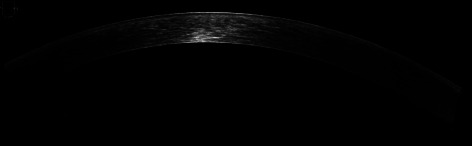
High-definition AS-OCT (Zeiss Cirrus 5000) of a normal cornea.

In line with previously reported study [17, 18], CT and ET acquisition was automated by the anterior segment spectral-domain Cirrus 5000 HD-OCT (Carl Zeiss Meditec, Germany). The cornea lens attachment was used for the pachymetry map and HD cornea scans (Figure 1). The subjects were positioned on the headrest with gaze fixed towards the fixation light target, and the image was centred on the pupil centre. Two scans were obtained for each eye by a single operator with 60-second rest periods to optimize the tear film rest and average values were registered. CT and ET mappings were obtained at a certain time, from 10 : 00 to 14 : 00, and at least 2 hours after awakening.

**Figure 2:**
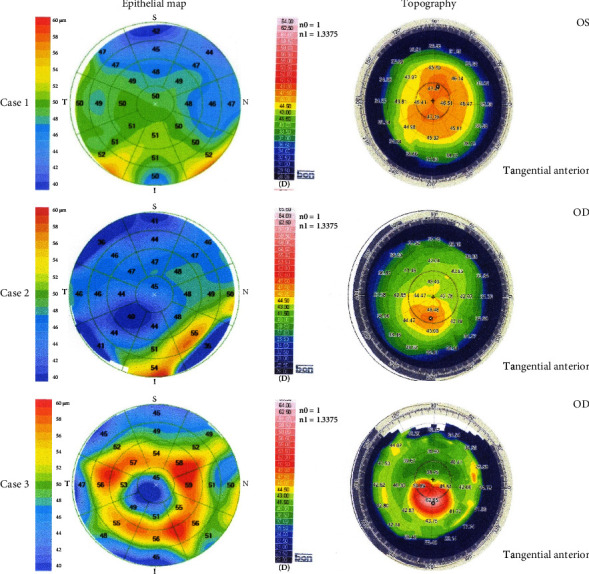
The epithelial and topography maps of normal eye, suspect keratoconus, and keratoconus eyes. The green circles overlaid on epithelial thickness maps had diameters of 2.0 mm, 5.0 mm, 7 mm, and 9 mm. The top row was a randomly chosen normal left eye of a 26-year-old female. The topographic simulated K readings were 45.87 D and 47.23 D. The Sirius software classifier provided a normal class. Min-Max ET (2_5) and SN-IT ET (2_5) had normal values (−6 *μ*m and −1 *μ*m, respectively). Case 2 (middle row) was a 20-year-old female with suspect keratoconus in the right eye. Her CDVA was 1.0. The simulated K values were 43.83 D and 50.01 D. The topography map showed inferior steepening. The ET map showed apical thinning inferotemporally. Min-Max ET (2_5) and SN-IT ET (2_5) exceeded cutoff values (−14 *μ*m and 8 *μ*m, respectively). Case 3 (third row) was a 32-year-old female with keratoconus in her right eye. Her CDVA was 0.2. The simulated K readings were 46.61 D and 48.64 D. The Sirius software classifier showed keratoconus class. The ET map showed apical thinning with surrounding thickening. Min ET (0_2), Min-Max ET (0_2), Min ET (2_5), Min-Max ET (2_5), and SN-IT ET (2_5) exceeded cutoff values of detecting keratoconus (37 *μ*m, −10 *μ*m, 38 *μ*m, −26 *μ*m, and 9 *μ*m, respectively). S = superior; T = temporal; I = inferior; N = nasal; K = keratometry; D = diopter; Min-Max = minimum-maximum; ET = epithelium thickness; SN-IT = superonasal-inferotemporal; *μ*m = micron; CDVA = corrected distance visual acuity; Min = minimum.

The pachymetry map scans include 8 radial scans (1024 axial scans each) repeated 5 times covering a 9 mm diameter area. The software algorithm measures ET as the distance between the middle of the first (tear film) and second (anterior surface of the Bowman layer) hyperreflective lines on the B-scan (Figure 2). CT was measured as the distance between the air-tear and cornea-aqueous interfaces. The pachymetry analysis tool provided automated cornea thickness measurement in seventeen sectors. Images were captured after the horizontal single scan line was placed on the corneal apex, where the hyperreflective corneal reflex was visible. Repeat scans were taken if the initial scan was decentred or had a poor corneal apex reflection.

Data was exported and processed with Cirrus HD-OCT review software (version 10.0) which provides average automated ET of four concentric ring-shaped zones around the centre of the cornea: 0–2 mm central, 2–5 mm paracentral, 5–7 mm midperipheral, and 7–9 mm peripheral, and CT from three concentric zones: 0–2 mm central, 2–5 mm paracentral, and 5–7 mm midperipheral. ET and CT were also presented for specific octants of the cornea: superior (S), inferior (I), temporal (T), nasal (N), superior nasal (SN), superior temporal (ST), inferior temporal (IT), and inferior nasal (IN) within the paracentral, midperipheral, and peripheral zones.

#### 2.1.1. Mean CT and ET Outcome Measures

CT and ET variables were registered at 0–2 mm central and 2–5 mm paracentral zones.

#### 2.1.2. Corneal Thickness Variables

Corneal thickness variables were defined as follows: Min CT (0_2): the minimum corneal thickness at the 0–2 mm central zone; Avg CT (0_2): the average corneal thickness at the 0–2 mm central zone; Max CT (0_2): the maximum corneal thickness at the 0–2 mm central zone; Min-Max CT (0_2): the minimum corneal thickness minus the maximum corneal thickness at the 0–2 mm corneal zone; Min CT (2_5): the minimum corneal thickness at the 2–5 mm paracentral zone; Avg CT (2_5): the average corneal thickness at the 2–5 paracentral zone; Max CT (2_5): the maximum corneal thickness at the 2–5 mm paracentral zone; Min-Max CT (2_5): the minimum corneal thickness minus the maximum corneal thickness at the 2–5 mm paracentral zone; S-I CT (2_5): the mean corneal thickness of the superior octant minus that of the inferior octant at the 2–5 mm paracentral zone; SN-IT CT (2_5): the difference between mean superonasal and mean inferotemporal corneal thickness at the 2–5 mm paracentral zone; CCT: the central corneal thickness.

#### 2.1.3. Epithelial Thickness Variables

Epithelial thickness variables were defined as follows: Min ET (0_2): the minimum epithelial thickness at the 0–2 mm central zone; Avg ET (0_2): the average epithelial thickness at the 0–2 mm central zone; Max ET (0_2): the maximum epithelial thickness at the 0–2 mm central zone; Min-Max ET (0_2): the minimum epithelial thickness minus the maximum corneal thickness at the 0–2 mm corneal thickness; Min ET (2_5): the minimum epithelial thickness at the 2–5 mm paracentral zone; Avg ET (2_5): the average epithelial corneal thickness at the 2–5 paracentral zone; Max ET (2_5): the maximum epithelial corneal thickness at the 2–5 mm paracentral zone; Min-Max ET (2_5): the minimum epithelial corneal thickness minus the maximum thickness at the 2–5 mm paracentral zone; S-I ET (2_5): the average epithelial thickness of the superior octant minus that of the inferior octant at the 2–5 mm paracentral zone; SN-IT ET (2_5): the difference between mean superonasal and mean inferotemporal corneal epithelial thickness at the 2–5 mm paracentral zone; CET: the thickness of the epithelium at the central point.

### 2.2. Statistical Analysis

Statistical analysis was performed using MedCalc Statistical Software, version 19.5.3 (MedCalc Software bv, Ostend, Belgium) and SPSS software (version 17, SPSS Inc., Chicago, IL, USA). One-way analysis of variance (ANOVA) with post hoc Bonferroni test was used. Receiver operating characteristic (ROC) curves were used to distinguish KC and SKC from N corneas. These curves were obtained by plotting sensitivity against 1-specificity, calculated for each value observed. The area under the ROC curve (AUC) measures discrimination, which is the ability of the test to accurately classify eyes with and without disease.

## 3. Results

One hundred and forty-four eyes were included and separated into three groups: N, SKC, and KC. Demographic characteristics for the respective groups are presented in Table 1. The N group included 65 eyes (63.07% female). The mean age was 27.07 ± 8.43 years. The mean sphere was −0.47 ± 2.70 dioptres (D), and the mean cylinder was −0.59 ± 1.61 D. The SKC group included 43 eyes (41.86% female). The mean patient age was 27.7 ± 6.82 years. The mean sphere was −0.11 ± 1.15 D, and the mean cylinder was −1.14 ± 1.26 D. The KC group included 36 eyes (47.22% female). The mean patient age was 26.49 ± 6.12 years. The mean sphere was −0.76 ± 1.88 D, and the mean cylinder was −2.72 ± 1.90 D. There was no statistically significant difference between the mean age of the three groups (ANOVA = 0.79). The mean sphere values were not significantly different between the three groups (ANOVA = 0.43). The mean cylinder values were highest in the KC group with statistically significant differences between the three groups (ANOVA < 0.0001).

The mean and standard deviation of OCT-based corneal thickness parameters for all groups are shown in Table 2. The mean minimum, average, and maximum corneal thickness values at the 2 mm central corneal zone were thinnest (444.94 ± 54.91 *μ*m, 469.41 ± 52.76 *μ*m, and 499 ± 48.96 *μ*m, respectively) in the KC group. The difference between the minimum and maximum corneal values at the central 2 mm zones was highest (−55.49 ± 20.94 *μ*m) in the KC group. All corneal thickness parameters obtained from the 2 mm central cornea were statistically significant different between the three groups (ANOVA < 0.05, for all). When comparing each of the two groups, all of the CT parameters reached statistical significance (*P*  <  0.05), except Max CT (0_2) which did not reveal any statistical significance between the KC and SKC groups (*P* = 0.227).

The minimum corneal thickness value at the 2–5 mm paracentral zone was lowest in the KC group (447.62 ± 49.38 *μ*m). The associations between opposite octants revealed that the highest differences between superior and inferior octants and between IT and SN octants were seen in the KC group (28.75 ± 24.05 *μ*m and 46.25 ± 24.72 *μ*m, respectively). While the difference between the minimum and maximum CT was almost similar in the N and SKC, the highest difference (−117.12 ± 44.84 *μ*m) was registered in the KC group. When comparing the three groups together, all CT parameters at the 2 mm paracentral cornea showed statistical significance (ANOVA < 0.05, for all). However, average and maximum CT values were not statistically significant between the KC and SKC groups (*P*  >  0.05, for both). S-I CT (2_5) only showed a statistically significant difference between the KC and N groups (*P* = 0.004). SN-IT CT (2_5) was statistically significance between the KC and N groups and between the KC and N groups, but not between the KC and SKC groups (*P* = 0.168). While the Min-Max CT (2_5) revealed statistical significance between the KC and N groups and between KC and SKC groups (*P* 0.0001, for both), the difference was not statistically significant between SKC and N groups (*P* = 0.0503)

ET parameter data are presented in Table 3. The minimum ET at the 2 mm central zone was thinnest (37.26 ± 4.51) *μ*m in the KC group. The highest difference (−13.62 ± 6.58 *μ*m) between the minimum and maximum ET at the central zone was registered in the KC group. All ET parameters obtained from the central cornea revealed statistically significant differences between the three groups (ANOVA < 0.0001), except Max ET (0_2), which did not show any statistical significance (ANOVA = 0.881). However, Min ET (0_2) and Min-Max ET (0_2) were not statistically different between the SKC and N groups (*P*  >  0.05, for both). The lowest ET value and the highest maximum ET value at the paracentral cornea were reported in the KC group (35.6 ± 4.48 *μ*m and 57.26 ± 8.77 *μ*m, respectively). In the same trend, the difference between minimum ET and maximum ET was highest (−21.59 ± 7.79 *μ*m) in the KC group. While the superior ET was thinner than the inferior ET in the N group, the superior ET was thicker than the inferior ET in the KC group. The highest difference between SN ET and IT ET (9.35 ± 4.7 *μ*m) was seen in the KC group. All ET parameters at the paracentral cornea were statistically significant between the three groups (ANOVA > 0.05), except Avg ET (2_5) which did not show any statistically significant difference (ANOVA = 0.183). CET was lowest in the KC (42.97 ± 5.38 *μ*m) with statistical significance between the three groups (ANOVA < 0.0001). SN-IT ET (2_5) and Min-Max (2_5) were the only ET parameters with statistically significant differences between SKC and N groups (*P* = 0.046 and *P* = 0.0004, respectively). Figure 2 shows the epithelial and topography maps of normal eye, suspect keratoconus, and keratoconus eyes.

Sensitivity, specificity, and area under the curve values identified by cutoff points of different CT parameter sets to differentiate eyes with SKC from N corneas and KC from N ones are presented in Table 4. To distinguish between SKC and controls, the highest AUC (0.76) was seen for CCT, with a sensitivity of 86.05% and a specificity of 55.7%. However, none of the CT parameters were strong enough to identify suspect keratoconus (AUC < 0.8, for all). While Min CT (0_2), Avg CT (0_2), Max CT (0_2), Min CT (2_5), Min-Max CT (2_5) SN-IT CT (2_5), and CCT were strong enough to distinguish between KC and N groups (AUC > 0.8, for all), the highest strength (0.94) was seen for Min CT (0_2). This parameter with a cutoff value of ≤−32 showed the highest sensitivity and specificity in detecting KC, at 94.12% and 93.4%, respectively.

Table 5 demonstrates the sensitivity, specificity, and area under the curve values identified by cutoff points of different ET parameter sets to differentiate eyes with SKC from N Corneas and KC from N ones. Min-Max ET (2_5) was the only ET parameter strong enough (AUC > 0.7) to differentiate between SKC and N eyes. This parameter with a cutoff value of ≤−9 had a sensitivity of 67.44% and a specificity of 76.7%.

In distinguishing between KC and N eyes, Min ET (0_2), Min-Max ET (0_2), SN-IT ET (2_5), Min-Max ET(2_5), and Min ET (2_5) were strong enough to detect KC eyes (AUC > 0.9, for all). However, the highest sensitivity (97.06%) was seen for Min-Max ET (2_5).

**Figure 3:**
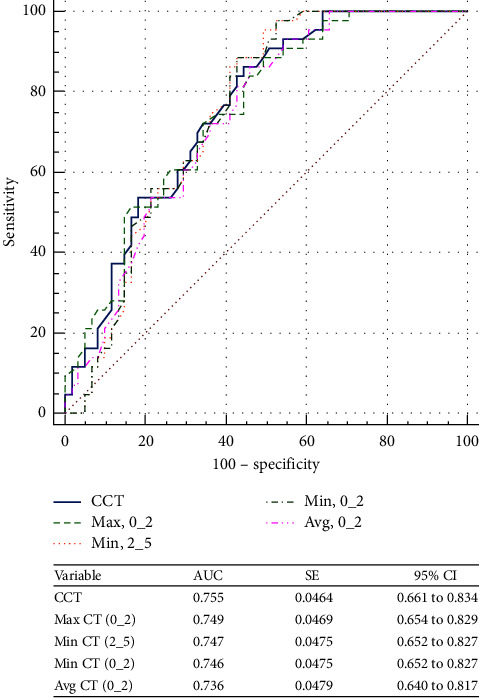
Comparison of corneal thickness parameters that showed the best area under the receiver operating characteristic curves to differentiate between suspect keratoconus and normal eyes. CCT = central corneal thickness; Max = maximum; Min = minimum; Avg = average; AUC = area under the receiver operating characteristic curve; SE = standard error; CI = confidence interval.

**Figure 4:**
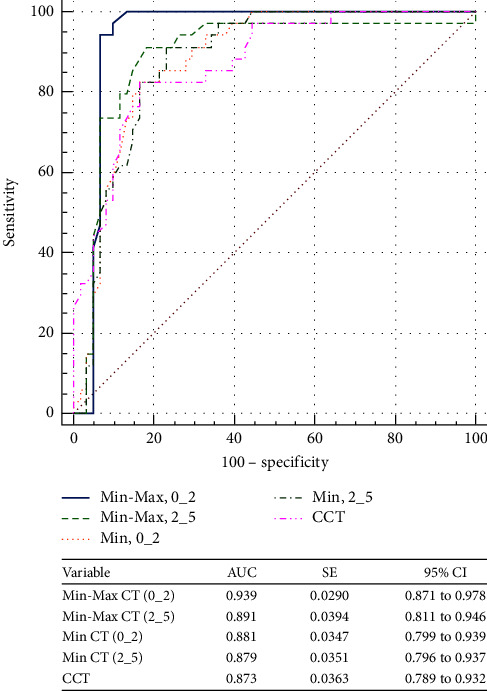
Comparison of the corneal thickness parameters that showed the best area under the receiver operating characteristic curves to differentiate between keratoconus and normal eyes. Min-Max = minimum-maximum; CT = corneal thickness; Min = minimum; CCT = central corneal thickness; AUC = area under the receiver operating characteristic curve; SE = standard error; CI = confidence interval.

**Figure 5:**
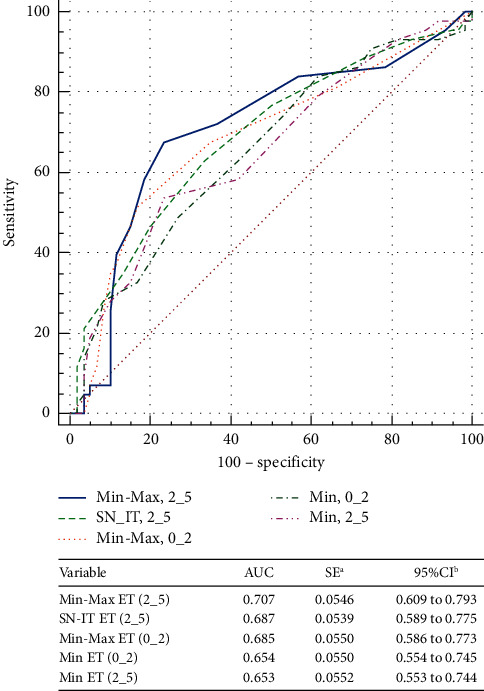
Comparison of the epithelial thickness parameters that showed the best area under the receiver operating characteristic curves to differentiate between suspect keratoconus and normal eyes. Min-Max = minimum-maximum; ET = epithelial thickness; SN-IT = superonasal-inferotemporal; Min = minimum; AUC = area under the receiver operating characteristic curve; SE = standard error; CI = confidence interval.

**Figure 6:**
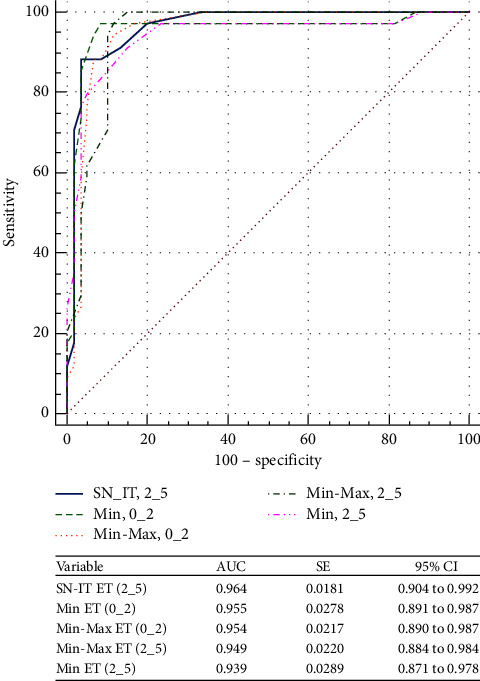
Comparison of the epithelial thickness parameters that showed the best area under the receiver operating characteristic curves to differentiate between keratoconus and normal eyes. SN-IT = superonasal-inferotemporal; ET = epithelial thickness; Min = minimum; Min-Max = minimum-maximum; AUC = area under the receiver operating characteristic curve; SE = standard error; CI = confidence interval.

Figures 3 and 4 show the ROC curves of the corneal thickness parameters to differentiate suspect KC from N eyes and KC from N eyes, respectively. While there was no CT parameter strong enough to distinguish SKC from N eyes (AUC < 0.8, for all), the same was not true for KC (AUC < 0.8, all). Min-Max CT (0_2) was the parameter with the best AUC to distinguish KC from N eyes, 0.939 (95% CI, 0.871–0.978). Figures 5 and 6 show the ROC curves of the different ET parameters to differentiate suspect KC from N eyes and KC from N eyes. While Min-Max ET (2_5) was the best ET parameter to differentiate between SKC and N eyes (AUC = 0.707; 95% CI, 0.609–0.793), SN-IT ET (2_5) was the best to differentiate between KC and N eyes (AUC = 0.964; 95% CI, 0.904–0.992).

## 4. Discussion

The aim of this study was to determine the diagnostic accuracy of CT and ET parameters measured by the anterior segment spectral-domain OCT (Zeiss Cirrus 5000 HD) in differentiating SKC, KC, from N corneas.

The results demonstrate the strong statistical significance of CT variables between KC and N eyes. To a lesser extent, SKC and N eyes comparisons showed significant differences for all CT variables except Min CT (2_5), S-I CT (2_5), and Min-Max CT (2_5). In their study, Hashemi et al. used high-resolution spectral-domain OCT (Heidelberg Engineering, Heidelberg, Germany) to investigate the diagnostic ability of total corneal thickness in detecting KC, where they found that CCT and Min-Median variables were the most sensitive indices for the diagnosis of KC [5]. In the current study, the two CT variables showing the greatest AUC to detect KC were Min-Max CT (0_2) and Min-Max CT (2_5); AUC = 0.94 and AUC = 0.89, respectively. Ambrosio et al. hypothesized that relative corneal thickness as opposed to unique thickness points is a more useful predictor in detecting KC and SKC [19]. Although stromal thickness progression variables showed the highest ability to detect KC, this was not the case for SKC eyes, as the highest AUC was seen for the thickness point variable (CCT; AUC = 0.76). Our findings are consistent with a previously reported study, where the authors found that CCT was the parameter with the highest ability to differentiate between subclinical KC and N eyes (AUC = 0.782) [18]. Reinstein et al. established that stromal thickness progression at a 2 mm radius from the thinnest point in N eyes was 29.9 ± 5.4, whereas it was 60.6 ± 25.6 *μ*m in KC eyes [14]. The data presented here show similar findings in the difference between the thinnest and thickest cornea at the 2 mm central zone at 27.92 ± 44.61 *μ*m in N eyes and 55.94 ± 20.94 *μ*m in KC eyes.

Coupling ET profiles with corneal tomography may further aid in screening for KC and may be useful in the clinical setting. Epithelial thickness data may allow for an earlier diagnosis of KC, as epithelial changes may precede any changes produced on the front surface of the cornea. Such ET changes in KC have been examined by other groups [20–25]. Fuente et al. evaluated total corneal thickness and corneal layers in 86 healthy young adults using SD-OCT, where they reported a central ET of 54.60 ± 4.25 *μ*m [26]. Erie et al. reported a central ET of 46.0 ± 5.0 *μ*m in N corneas measured by confocal microscopy [27]. Their measurements, which excluded the precorneal tear film thickness, were thinner than the central ET presented in this report of 49.92 ± 3.98 *μ*m in N eyes. Of note, the measurements in this study included the thickness of the tear film, estimated at 3 *μ*m, which is likely to account for the difference. Li et al. used a Fourier-domain OCT system (RTVue, Optovue Inc., Fremont, CA, USA) to measure corneal epithelium thickness over a 6 mm diameter. The central epithelial thickness of N eyes from their study, which included the tear film, was 52.3 ± 3.6 *μ*m. Moreover, the corneal epithelial thickness was demonstrated to be thicker inferiorly by −1.6 *μ*m in the N corneas in comparison with the superior cornea [21]. The data findings presented here concur with those of Li and colleagues as demonstrated by an S-I mean difference of −1.5 *μ*m.

Previous studies have shown that the apex of early KC focal steepening is compensated by corneal epithelial thinning, and the degree of potential epithelial remodelling is dependent on the severity of the keratoconus [20]. In this study, all ET variables showed statistically significant differences between KC and N eyes. The epithelial doughnut pattern described by Reinstein et al. consists of a localized zone of thinning surrounded by an annulus of thickened epithelium. This pattern was characteristic of all keratoconic eyes. Reinstein et al. also demonstrated that the mean thinnest epithelium in KC eyes was 38.2 ± 5.8, whereas the mean thickest epithelium was 66.8 ± 7.2 *μ*m with a mean difference between the thinnest and thickest epithelium of 28.6 ± 10.8 *μ*m. Our corresponding values were 35.68 ± 4.48 *μ*m, 57.26 ± 8.77 *μ*m, and 21.59 ± 7.79 *μ*m, respectively. These values were registered at the paracentral zone where the cone is more pronounced. In their study investigating epithelial thickness distribution characteristics in keratoconic eyes, Kanellopoulos and Asimellis found that the epithelium was thinner at the inferotemporal area in comparison with the superonasal area and thinner inferiorly [23]. In agreement with these results, we found that the epithelium in the keratoconic eyes was thinner inferiorly than superiorly by 6.62 ± 9.16 *μ*m and thinner inferotemporally than superonasally by 9.35 ± 4.7 *μ*m. The opposite held true in the N group, the epithelium was thinner superiorly than inferiorly and thinner superonasally than inferotemporally. This phenomenon can be justified by the mechanical chaffing and abrasion caused by the accelerated movement of the upper tarsus with a larger force being applied on the superior epithelium [28].

In their study, Li et al. found that root-mean-square pattern deviation (RMSPD) was the only epithelial thickness-based variable with excellent diagnostic power to differentiate between KC and N eyes, while the other variables varied from poor (central, superior, and inferior zonal epithelial thicknesses), to fair (S-I), to good (Min ET and Min-Max ET) [21]. In contrast, several corneal epithelial thickness-based variables evaluated in this study showed excellent (Min ET (0_2), Min-Max ET (0_2), SN-IT ET (2_5), Min-Max ET (2_5), and Min ET (2_5) minimum, AROC > 0.9, for all) diagnostic power in differentiating KC from N eyes. The difference in the diagnostic power of ET between the two studies may be due to the differences in ET variables obtained in each study, although both studies obtained their ET variables from the 5 mm central cornea. For example, the SN-IT ET (2_5) variable was not included in the Li et al. study, despite it proving to be one of the more favourable variables in discriminating between the KC group and the N group (AUC = 96; sensitivity = 88.24%; specificity = 96.7%).

Ostadian et al. found that the central epithelium was significantly thinner in subclinical KC corneas than in N corneas [24]. In this study, the central ET was not significantly different between SKC and N eyes. However, SN-IT ET (2_5) and Min-Max (2_5), were the only ET variables with statistical significance between SKC and N eyes, with the highest ability (AUC = 0.71) for Min-Max ET (2_5) to differentiate between SKC and N eyes.

Our findings demonstrated that ET variables were superior to CT variables in differentiating between KC and N eyes, since only one CT variable (Min-Max (0_2)) revealed excellent diagnostic power (AUC > 0.9) to detect KC eyes, while five ET variables (Min ET (0_2), Min-Max ET (0_2), SN-IT ET (2_5), Min-Max ET (2_5), and Min ET (2_5)) were excellent in diagnosing KC eyes.

Diagnosing early-stage KC remains a challenge. Our results showed that both CT and ET variables derived from OCT imaging were not sufficient to differentiate between SKC and N eyes (AUC < 0.8, for both). Estrada and Alio used Placido/Sheimpflug-based corneal tomography (Sirius) to investigate the diagnostic power of posterior corneal surface parameters in differentiating KC from N eyes, where they found that root-mean-square per unit of area (RMS/A) and keratoconus vertex back (KVb) were the variables with the highest discriminating capabilities between normal and mild KC cases with an area under the curve of 0.96 and 0.97, respectively [29]. In our previously reported study, symmetry index back (SIb) derived from the posterior corneal surface was the parameter with the highest diagnostic power to detect SKC eyes [13]. Consistent with these results, Heidari et al. found that Sirius SIb was the parameter with the highest power to distinguish subclinical KC from N eyes (AUC = 0.908) [30]. However, this was not unexpected, since changes in the posterior corneal surface may be one of the first clinically detectable signs of KC. In contrast, when we investigated the application of anterior and posterior corneal higher-order aberrations (HOAs) in detecting KC and SKC, coma (3, ±1) derived from the anterior corneal surface was the parameter with the highest ability to discriminate between SKC and N eyes (AUC = 0.922; cutoff > 0.2) [15]. While Estrada and Alio found that posterior corneal HOAs were useful to differentiate normal from mild KC cases, our results demonstrated that posterior corneal HOA parameters were unsatisfactory in discriminating between SKC and normal eyes [29].

One limitation of this study is that pachymetric and epithelial thickness maps were confined to the central 5 mm diameter of the cornea. The cone apex is located in the central 5 mm diameter of the cornea in the vast majority of KC eyes [19]. Thus, we opted to evaluate the characteristics of these variables in the 2 mm central and 2–5 mm paracentral corneas. OCT measurements in our study included the thickness of the precorneal tear film. Tear film thickness values range between 3 and 5 *μ*m [27, 28]. OCT epithelial and corneal measurements including the tear film thickness may lead to potential inaccuracies of the absolute values and this could be considered a limitation of the study. The small sample size is another limitation in this study, as using small sample sizes may yield unreliable results. Increasing the sample size and using a power calculation in future studies will be key to limit the risk of sampling bias.

In conclusion, the cutoff points proposed in our study had sufficient sensitivity and specificity to differentiate between KC and N eyes. In SKC, however, the use of a single parameter to diagnose KC is insufficient. The combination of OCT-derived data with posterior corneal surface and wavefront data to improve automatic recognition of early KC will be the target of our future research. Nevertheless, epithelial and corneal thickness mapping is a vital tool for the refractive surgeon, with particular strengths in the early detection of keratoconus and monitoring of ectasia due to its predictable transformation in pathological and ectatic conditions as highlighted in this study.

## Figures and Tables

**Table 1:** Demographic characteristics of the study groups.

	Normal	SKC	KC	ANOVA	*P* values		
Patients (*n*)	65	43	36		SKC vs. normal	KC vs. normal	KC vs. SKC
Sex							
F	41	18	17				
M	24	25	19				
Age (years) (mean ± SD)	27.03 ± 8.43	27.7 ± 6.82	26.49 ± 6.12	0.79			
Sphere (D) (mean ± SD)	−0.47 ± 2.70	−0.11 ± 1.15	−0.76 ± 1.88	0.43			
Cylinder (D) (mean ± SD)	−0.59 ± 1.61	−1.14 ± 1.26	−2.72 ± 1.90	**<0.0001**	0.26	**<0.0001**	**<0.0001**

SKC = suspect keratoconus; KC = keratoconus; *n* = number; F = female; M = male; yr = years old; D = diopter; SD = standard deviation. Statistically significant values (*P*  <  0.05). Values in bold are statistically significant.

**Table 2:** OCT-based corneal thickness data in normal, suspect keratoconus, and keratoconus groups.

Pachymetry (*μ*m)	Normal				SKC				KC				ANOVA	*P* values		
	Mean	SD	Range		Mean	SD	Range		Mean	SD	Range			SKC vs. normal	KC vs. normal	KC vs. SKC
Min CT (0_2)	524.97	60.81	294	645	491.49	30.92	422	546	444.94	54.91	287	527	**<0.0001**	**0.004**	**<0.0001**	**<0.0001**
Avg CT (0_2)	538.02	44.62	456	655	502.21	31.21	436	560	469.41	52.76	307	559	**<0.0001**	**<0.0001**	**<0.0001**	**0.004**
Max CT (0_2)	552.89	42.34	480	671	516.91	32.23	452	579	499.94	48.96	344	592	**<0.0001**	**<0.0001**	**<0.0001**	0.227
Min-Max (0_2)	−27.92	44.61	−256	−8	−25.42	10.72	−58	−9	−55.94	20.94	−106	−26	**0.0001**	**0.0015**	**<0.0001**	**0.0001**
Min CT (2_5)	524.49	63.19	282	644	491.58	30.8	422	546	447.62	49.38	312	527	**<0.0001**	**0.005**	**<0.0001**	**0.001**
Avg CT (2_5)	556.66	43.25	483	671	523.56	32.28	460	581	510.65	37.27	401	586	**<0.0001**	**<0.0001**	**<0.0001**	0.444
Max CT (2_5)	598.46	45.73	515	718	564.51	36.12	503	655	564.74	32.72	505	623	**<0.0001**	**<0.0001**	**<0.0001**	1
S-I CT (2_5)	6.80	40.07	−214	59	18.7	15.12	−5	76	28.75	24.05	−38	90	**0.0041**	0.157	**0.004**	0.482
SN-IT CT (2_5)	19.51	36.12	−179	63	33.49	15.11	−2	80	46.25	24.72	−30	109	**0.0001**	**0.043**	**<0.0001**	0.168
Min-Max CT (2_5)	−73.97	52.32	−345	−39	−72.93	20	−133	−42	−117.12	44.84	−220	7	**0.0001**	0.0503	**0.0001**	**0.0001**
CCT	537.13	43.66	449	652	498.93	31.42	433	558	462.03	55.99	287	554	**<0.0001**	**<0.0001**	**<0.0001**	**0.001**

Min = minimum; CT = corneal thickness; Avg = average; Max = maximum; Min-Max = minimum-maximum; S-I = superior-inferior; SN-IT = superonasal-inferotemporal; CCT = central corneal thickness; SKC = suspect keratoconus; KC = keratoconus; SD = standard deviation. Statistically significant values (*P*  <  0.05). Values in bold are statistically significant.

**Table 3:** OCT-based epithelial thickness data in normal, suspect keratoconus, and keratoconus groups.

Epithelial thickness (*μ*m)	Normal				SKC				KC				ANOVA	*P* values		
	Mean	SD	Range		Mean	SD	Range		Mean	SD	Range		*P* value	SKC vs. normal	KC vs. normal	KC vs. SKC
Min ET (0_2)	47.57	4.1	33	58	45.74	4.25	38	58	37.26	4.51	30	52	**<0.0001**	0.097	**<0.0001**	**<0.0001**
Avg ET (0_2)	49.66	3.71	38	60	48.47	4.4	41	63	43.71	4.93	36	57	**<0.0001**	0.486	**<0.0001**	**<0.0001**
Max ET (0_2)	51.34	3.88	44	62	50.91	4.61	43	66	50.88	7.5	42	81	0.8813			
Min-Max ET (0_2)	−3.77	3.17	−20	−1	−5.16	2.74	−11	−1	−13.62	6.58	−39	−4	**<0.0001**	0.288	**<0.0001**	**<0.0001**
Min ET (2_5)	44.45	3.56	33	54	42.84	3.51	37	55	35.68	4.48	29	48	**<0.0001**	0.106	**<0.0001**	**<0.0001**
Avg Et (2_5)	48.75	3.38	42	60	48.56	3.77	42	59	47.21	5.23	39	63	0.1837			
Max ET (2_5)	52.6	4.7	45	69	53.3	5.15	43	69	57.26	8.77	47	94	**0.0016**	1	**0.001**	**0.016**
S-I ET (2_5)	−1.55	3.14	−6	11	0.74	8.38	−9	50	6.62	9.16	−4	48	**<0.0001**	0.289	**<0.0001**	**0.001**
SN-IT ET (2_5)	−0.1	3	−8	14	1.72	3.73	−10	10	9.35	4.7	1	19	**<0.0001**	**0.046**	**<0.0001**	**<0.0001**
Min-Max (2_5)	−8.15	4.65	−26	−3	−10.47	4.18	−22	−4	−21.59	7.79	−52	−11	**<0.0001**	**0.0004**	**<0.0001**	**<0.0001**
CET	49.92	3.98	36	60	48.49	4.88	41	63	42.97	5.38	33	55	**<0.0001**	0.373	**<0.0001**	**<0.0001**

Min = minimum; ET = epithelial thickness; Avg = average; Max = maximum; Min-Max = minimum-maximum; S-I = superior-inferior; SN-IT = superonasal-inferotemporal; CET = central epithelial thickness; SKC = suspect keratoconus; KC = keratoconus; SD = standard deviation. Statistically significant values (*P*  <  0.05). Values in bold are statistically significant.

**Table 4:** Sensitivity, specificity, and area under the curve values identified by cutoff points of different OCT-based corneal thickness parameter sets to differentiate eyes with suspect keratoconus from normal corneas and keratoconus from normal ones.

Pachymetry (*μ*m)	Cutoff value		AUC		Sensitivity (%)		Specificity (%)	
	Normal vs. SKC	Normal vs. KC	Normal vs. SKC	Normal vs. KC	Normal vs. SKC	Normal vs. KC	Normal vs. SKC	Normal vs. KC
Min CT (0_2)	≤526	≤478	0.75	0.88	88.37	82.86	57.4	83.6
Avg CT (0_2)	≤535	≤497	0.74	0.85	86.05	82.35	54.1	78.7
Max CT (0_2)	≤557	≤526	0.75	0.8	88.37	76.47	50.8	73.8
Min-Max CT (0_2)	≤−26	≤−32	0.68	0.94	53.49	94.12	86.9	93.4
Min CT (2_5)	≤537	≤493	0.75	0.88	95.35	91.18	50.8	77.1
Avg CT (2_5)	≤581	≤542	0.73	0.78	100	91.18	36.1	60.7
Max CT (2_5)	≤613	≤608	0.72	0.72	90.7	91.18	49.2	50.8
Min-Max CT (2_5)	≤−77	≤−77	0.61	0.89	41.86	91.18	41.9	82
S-I CT (2_5)	>1	>15	0.58	0.77	95.35	90.62	23	59
SN-IT CT (2_5)	>25	>35	0.66	0.83	74.42	78.12	50.8	82
CCT	≤533	≤491	0.76	0.87	86.05	82.35	55.7	83.6

Min = minimum; CT = corneal thickness; Avg = average; Max = maximum; Min-Max = minimum-maximum; S-I = superior-inferior; SN-IT = superonasal-inferotemporal; CCT = central corneal thickness; AUC = area under the receiver operating characteristic curve; SKC = suspect keratoconus; KC = keratoconus.

**Table 5:** Sensitivity, specificity, and area under the curve values identified by cutoff points of different epithelial thickness parameter sets to differentiate eyes with suspect keratoconus from normal corneas and keratoconus from normal ones.

Epithelial thickness (*μ*m)	Cutoff value		AUC		Sensitivity (%)		Specificity (%)	
	Normal vs. SKC	Normal vs. KC	Normal vs. SKC	Normal vs. KC	Normal vs. SKC	Normal vs. KC	Normal vs. SKC	Normal vs. KC
Min ET (0_2)	≤48	≤42	0.65	0.96	83.72	94.12	37.7	93.4
Avg ET (0_2)	≤48	≤47	0.63	0.85	58.14	85.29	65.6	75.4
Max ET (0_2)	≤49	≤49	0.56	0.6	51.16	52.94	68.9	68.9
Min-Max ET (0_2)	≤−5	≤−6	0.69	0.96	51.16	94.12	83.6	88.5
Min ET (2_5)	≤48	≤41	0.65	0.94	83.72	91.18	37.7	85
Avg ET (2_5)	≤48	≤46	0.63	0.65	58.14	55.88	65.6	76.7
Max ET (2_5)	≤49	>54	0.56	0.7	51.16	58.82	68.9	73.3
Min-Max ET (2_5)	≤−9	≤−12	0.71	0.95	67.44	97.06	76.7	88.3
S-I ET (2_5)	>0	>1	0.63	0.88	39.53	76.47	85	91.7
SN-IT ET (2_5)	>0	>4	0.69	0.96	62.79	88.24	66.7	96.7
CET	≤48	≤43	0.63	0.85	55.81	55.88	65.6	98.4

Min = minimum; ET = epithelial thickness; Avg = average; Max = maximum; Min-Max = minimum-maximum; S-I = superior-inferior; SN-IT = superonasal-inferotemporal; CET = central epithelial thickness; AUC = area under the receiver operating characteristic curve; SKC = suspect keratoconus; KC = keratoconus. Criteria of all variables had units of *μ*m.

## Data Availability

The data that support the findings of this study are available from the corresponding author upon reasonable request. The data are not publicly available due to privacy or ethical restrictions.
